# Supplementary material for: Causal interplay between lactose intolerance and gut microbiota: a combined bidirectional Mendelian randomization and in vivo validation study
Source: Front Nutr. 2026 Jun 1;13:1803337. doi: 10.3389/fnut.2026.1803337 (PMC13265576; doi:10.3389/fnut.2026.1803337)
Supplement: Supplementary file 1 [file Data_Sheet_1.zip › supplementary materials/Forward/leave one out cross validation/ebi-a-GCST90027630.finngen_R12_E4_LACTONAS.pdf]

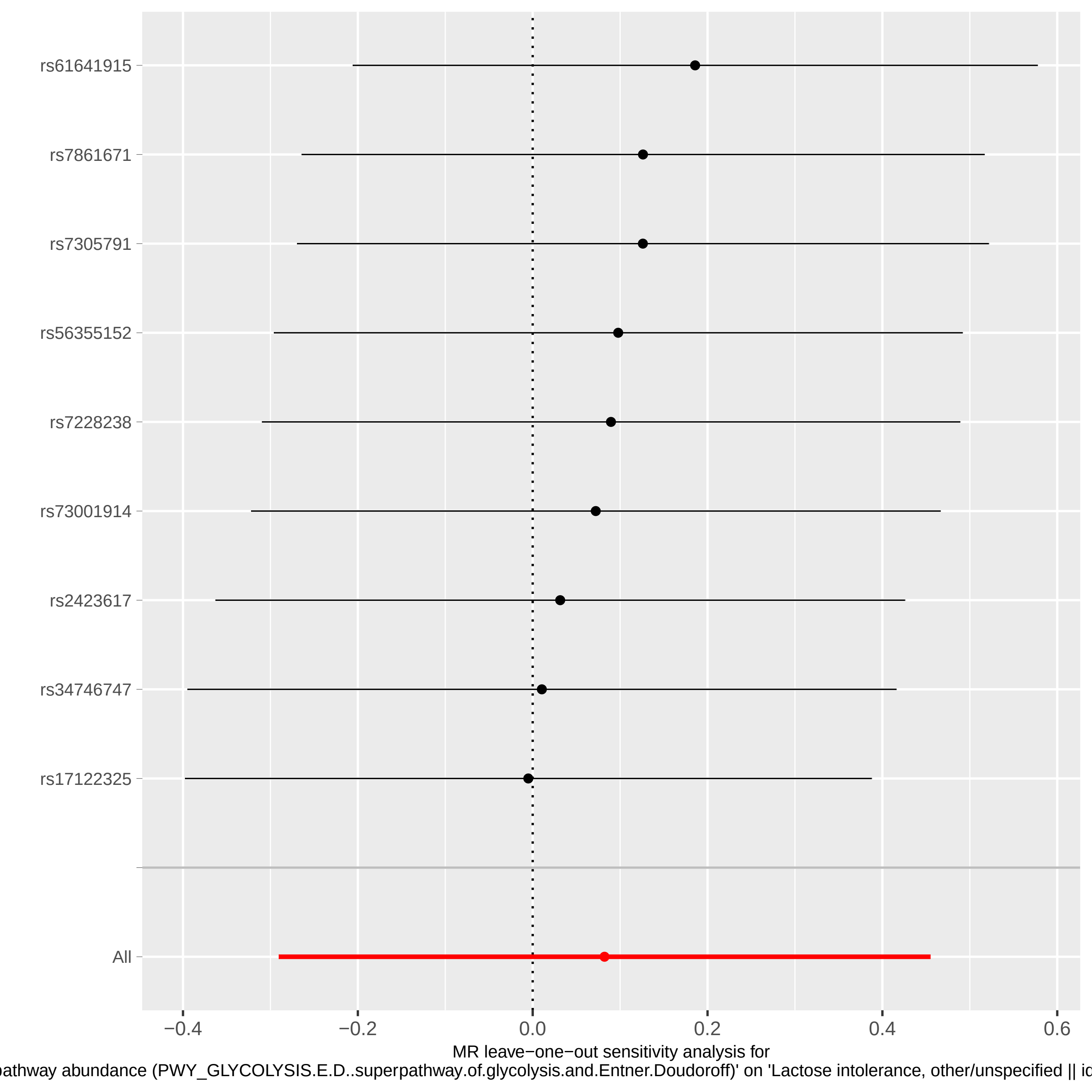
rs61641915
rs7861671
rs7305791
rs56355152
rs7228238
rs73001914
rs2423617
rs34746747
rs17122325
All
−0.4
−0.2
0.0
0.2
0.4
0.6
MR leave−one−out sensitivity analysis for
pathway abundance (PWY_GLYCOLYSIS.E.D..superpathway.of.glycolysis.and.Entner.Doudoroff)' on 'Lactose intolerance, other/unspecified || id
